# Lung ultrasound: a useful tool in the weaning process?

**DOI:** 10.5935/0103-507X.20160002

**Published:** 2016

**Authors:** Fabiola Prior Caltabeloti, Jean-Jacques Rouby

**Affiliations:** 1 Department of Anesthesiology, Hospital das Clínicas, Faculdade de Medicina, Universidade de São Paulo - São Paulo (SP), Brazil.; 2 Departament of Anesthesiology and Critical Care Medicine, Pitié-Salpêtrière Hospital, University Pierre and Marie Curie of Paris 6, Paris, France.

## INTRODUCTION

The incidence of pulmonary complications related to mechanical ventilation is an important issue among critically ill patients. Reducing the duration of respiratory support is essential for minimizing these complications. The extubation of a patient marks the end of the weaning process. Unfortunately, even after a successful spontaneous breathing trial (SBT), approximately 30% of patients develop respiratory distress within 48 hours of extubation; this results in extubation failure and requires either therapeutic non-invasive ventilation or reintubation.^(1)^ The loss of pulmonary aeration following extubation is a hallmark of extubation failure, leading to impaired gas exchange, prolonged mechanical ventilation, and increased morbidity and mortality.^(2)^ The pathophysiology is multifactorial.

The amount of lung aeration loss can be quantified via lung ultrasound during different clinical conditions including the weaning process. It is a non-invasive and radiation-free procedure, which can be performed quickly at the bedside and enables a dynamic assessment of lung aeration changes depending on ventilation conditions, as opposed to a chest x-ray. For many years, lungs were not considered accessible by ultrasound because air does not allow for the transmission of ultrasound waves. However, the artifacts produced at the interface between the lungs and fluids, for example, can be easily identified by lung ultrasound.

**Figure 1:**
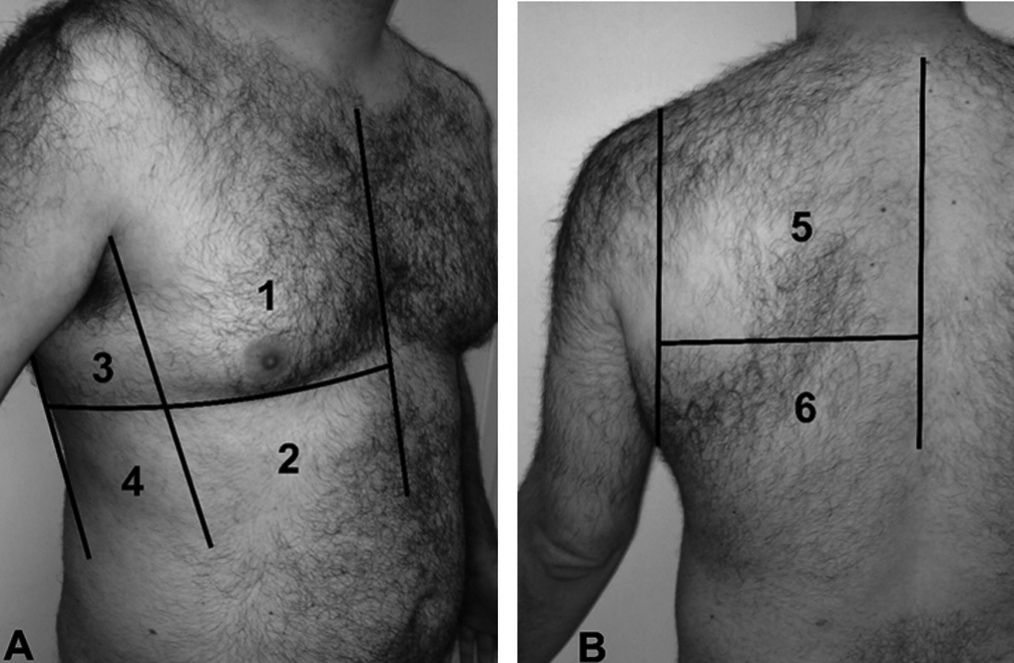
Lung ultrasound score.

Lung aeration loss can be estimated using by a validated score called the Lung Ultrasound Score (LUS). As previously recommended,^(3-5)^ all of the intercostal spaces of the anterior, lateral and posterior regions of both lungs (6 regions per side) are evaluated (Figure 1). For each region, the worst ultrasound pattern is considered to be representative of the entire region. Normal aeration is represented by the presence of lung sliding and horizontal A lines, or less than 3 vertical B lines; a score of 0 is assigned to a lung region if all of the intercostal spaces show normal aeration. A moderate loss of aeration is characterized by multiple regularly or irregularly spaced B lines that originate from pleural line or from small juxta-pleural consolidations; a score of 1 is assigned to a lung region if all of the intercostal spaces show a moderate loss of aeration. Severe loss of aeration is characterized by the presence of coalescent B lines in several intercostal spaces, occupying the whole intercostal space; a score of 2 is assigned to the examined region. Complete loss of lung aeration, as observed in lung consolidation, is characterized by tissue echogenicity with static or dynamic air bronchograms; a score of 3 is assigned to the examined region. The scores of the 12 examined regions are summed to calculate the LUS score, which ranges between 0 and 36. Video files and detailed ultrasound patterns that characterize the different stages of aeration can be freely downloaded by visiting http://www.reapitie-univparis6.aphp.fr and clicking on the "Basic skills in lung ultrasound" link.

## LUNG ULTRASOUND IN GENERAL CLINICAL PRACTICE

Alveolar recruitment resulting from the administration of positive end-expiratory pressure in patients with acute respiratory distress syndrome (ARDS)^(4)^ and after recovery from ventilator-associated pneumonia during antibiotic treatment can be successfully assessed by lung ultrasound.^(5)^ The evolution of ARDS can also be monitored using lung ultrasound.^(6)^

Lung ultrasonography has also been validated as a sensitive tool for assessing the risk-benefit ratio of fluid loading in patients with septic shock and ARDS. While hemodynamic parameters and oxygenation improved in the study sample, the patients' LUSs increased, indicating aeration loss. Therefore, the use of lung untrasonography may prevent fluid overload.^(7)^

Recently, Soummer et al.^(8)^ showed that a LUS < 13 at the end of a SBT is predictive of extubation success. On the other hand, a LUS > 17 is highly predictive of postextubation distress and extubation failure. Lung derecruitment during the SBT in patients who later experienced extubation failure mainly comprised partial loss of lung aeration rather than new consolidation. This finding suggests that prophylactic non-invasive ventilation (NIV) can prevent derecruitment. A recent multicenter randomized controlled trial demonstrated that high flow nasal oxygen (HFNO) is as efficient as NIV in preventing reintubation in cardiac patients with severe postoperative respiratory failure.^(9)^ Another multicenter randomized controlled trial reported that compared to a combination of NIV and HFNO, continuous HFNO is more effective at preventing intubation in severely hypoxemic patients who were admitted to the intensive care unit (ICU) for acute respiratory distress caused by community acquired pneumonia.^(10)^ This positive effect has been shown to be associated with a reduction in mortality.^(11)^ Interestingly, a randomized monocenter study found that HFNO is much more effective than conventional oxygen therapy in preventing extubation failure during the weaning process.^(12)^ In addition, it appears to be much more comfortable for patients.

## IMPLEMENTATION OF LUNG ULTRASOUND IN THE WEANING PROCESS

Based on these different studies, we designed a multicenter randomized controlled trial, the WEANLUS Brazil Study, to assess whether the continuous administration of HFNO prevents extubation failure in patients categorized as at-risk based on a LUS > 13 at the end of a successful SBT. The trial is now underway and will include 640 patients who were older than 18 years and mechanically ventilated for more than 48 hours. Patients will be divided in two groups after extubation: the control group and intervention group. A LUS will be calculated for each patient at the end of their successful SBT. In the control group, the physician in charge of the patient will be blinded to the LUS, and all of the patients will receive conventional oxygen therapy after extubation. NIV will be administered to the patients with well-defined criteria of severe respiratory failure, and they will be classified as experiencing "extubation failure". In the intervention group, the physician in charge of the patient will not be blinded to the LUS. HFNO will be administered only to the patients at a high risk of extubation failure (i.e., patients with a LUS > 13 at the end of their SBT). The goal of the study is to show a 30% decrease in the incidence of extubation failure, an increase in the number of days without mechanical ventilation (invasive and non-invasive) from randomization, a decrease in the length of stay in the ICU and a reduction in mortality at D30 and D90 in the high-risk group (LUS >13). Furthermore, patient comfort under each technique of oxygenation (conventional O_2_ and HFNO) will be assessed by assigning a comfort score based on a visual numerical scale.
